# Telerehabilitation on the Physical and Functional Capacity of Traumatic Fractures of the Upper Limbs: A Systematic Review with Meta-Analysis

**DOI:** 10.63144/ijt.2025.6667

**Published:** 2025-06-12

**Authors:** Guilherme Grivicich da Silva, Francisco Xavier de Araùjo, André Hillebrand Andriola, Gustavo Costa Pereira, Marcelo Faria Silva

**Affiliations:** 1 Rehabilitation Service, Hospital Cristo Redentor, Grupo Hospitalar Conceição (GHC), Porto Alegre, Rio Grande do Sul, Brazil; 2 Physical Therapy Department, Federal University of Pelotas, Pelotas, Rio Grande do Sul, Brazil; 3 Physical Therapy, Health School, University of Vale do Rio dos Sinos (UNISINOS), São Leopoldo, Rio Grande do Sul, Brazil; 4 Physical Therapy Department, Federal University of Health Sciences of Porto Alegre (UFCSPA), Porto Alegre, Rio Grande do Sul, Brazil

**Keywords:** Fractures, Functional capacity, Physical capacity, Telerehabilitation

## Abstract

Traumatic injuries to the upper limbs, especially to the hands and wrists, have the potential to trigger chronic conditions with pain, loss of productivity and decreased quality of life. The aim of this study was to systematically review the literature on the effects of telerehabilitation on the physical and functional capacity of individuals with traumatic upper limbs fractures. Searches were conducted in the MEDLINE/PubMed, EMBASE, PEDRO, Cochrane, LILACS, and Science Direct databases. Three randomized clinical trials involving 830 patients with at least one intervention group and one comparison group were included in this systematic review. Risk of bias was assessed using the PEDro Scale and the certainty of the evidence was assessed using GRADE. Telerehabilitation seems to have favorable effects on functional capacity and pain perception and controversial effects on physical capacity (handgrip strength) in individuals with traumatic upper limb fractures.

Traumatic injuries to the upper limbs, particularly the hands and wrists, can lead to chronic conditions characterized by pain, decreased productivity, and a diminished quality of life (Robinson et al., 2016). While the incidence of these injuries has been declining in countries with higher socioeconomic development, regions with lower and middle socioeconomic indices have witnessed an increasing rate over the past 30 years (Crowe et al., 2020). Upper limb bone trauma occurs globally with a high frequency; however, the resulting functional impairment and disability vary depending on the severity of the injury, timely diagnosis, and effective treatment (Crowe et al., 2020).

For humerus fractures, there is insufficient evidence from randomized controlled trials (RCTs) to inform choices between different nonsurgical, surgical, or rehabilitation interventions (Handoll et al., 2022). Telerehabilitation was initially developed for hospitalized patients to facilitate earlier discharge, reducing hospitalization time and costs for both patients and healthcare providers (Peretti et al., 2017). The adaptability and flexibility of telerehabilitation can mitigate impairments and disabilities (Carey et al., 2007). Despite promising evidence supporting the effectiveness of telerehabilitation initiatives, challenges and barriers to implementation are complex, multifaceted, and context-dependent (Baroni et al., 2023). Telerehabilitation is a relatively new and promising service delivery model but currently lacks standardized procedures or protocols. Different telerehabilitation modalities are being evaluated in a limited number of patients with diverse clinical conditions (Peretti et al., 2017).

In the synchronous modality (real-time), telerehabilitation appears to be superior to in-person treatment for improving physical condition in various musculoskeletal conditions, including low back pain, total knee and hip arthroplasty (Cottrell et al., 2017), and is considered a viable option for clinical management (Cottrell & Russell, 2020). Moreover, the management of common musculoskeletal conditions through telerehabilitation can lead to comparable levels of patient satisfaction as conventional in-person treatments, offering reliable assessment and effective treatment (Bucki et al., 2021). Telerehabilitation via mobile applications has demonstrated potential positive effects on self-efficacy, patient-reported physical capacity, health-related quality of life, and levels of anxiety and depression (Wang et al., 2023). Beyond orthopedic disorders, telerehabilitation can be considered an alternative for health education and lifestyle transformation (Baroni et al., 2023). A systematic review revealed that telerehabilitation-based physical therapy assessments are technically feasible for measuring pain, edema, range of motion, muscle strength, balance, gait, and functional outcomes with good global concurrent validity (Mani et al., 2017).

Therefore, there is a need to understand the current state of the art in telerehabilitation for traumatic upper limb fractures. The objective of this research was to systematically review the effects of telerehabilitation on the physical and functional capacity of individuals with traumatic upper limb fractures.

## Methods

This systematic review adhered to the Preferred Reporting Items for Systematic Reviews and Meta-Analyses (PRISMA) guidelines (Liberati et al., 2009) and was prospectively registered in the International prospective register of systematic reviews (PROSPERO registration number: CRD42023474417).

### Search Strategies

The following databases were searched from their inception to July 31, 2023: Cochrane CENTRAL, PubMed, LILACS, PEDro, Science Direct, and Embase. Search terms used individually or in combination, including MeSH and its Entree Terms, were: "telerehabilitation," "telemedicine," and "bone fractures." To enhance search sensitivity, terms related to specific outcomes of interest were not included.

### Study Selection

Only RCTs with at least one intervention group and one comparison group were included. The intervention group must have received some form of telerehabilitation, while the control group underwent conventional in-person physiotherapy or a health education strategy. Quasi-randomized trials, non-randomized trials, single-arm clinical trials, abstracts, and conference presentations were excluded. No restrictions were imposed on language, publication date, patient gender, or ethnicity.

Functional capacity was defined in this review as the ability to perform activities necessary for self-care and independent living. Its measurement can be achieved using validated functionality scales commonly employed in the scientific community (e.g., Functional Independence Measure). Physical capacity encompasses outcomes related to range of motion, strength, or balance, assessed using any valid and reliable outcome measure (e.g., goniometer, strength tests).

Two reviewers (GGS and AHA) independently screened titles and abstracts of the initial search. A standardized screening checklist with eligibility criteria was applied to each study. Studies that did not meet the criteria based on titles and abstracts were excluded. Full-text versions of the remaining studies, along with those that raised doubts during the initial screening, were independently evaluated again by two reviewers to determine eligibility. In cases of disagreement, a third reviewer (MFS) was consulted. Studies with insufficient information to determine eligibility had their authors contacted by email for further clarification. If clarification was not obtained, the study was excluded.

### Data Extraction and Risk of Bias Assessment

Two reviewers (GCP and AHA) independently extracted data from the included studies. Disagreements regarding study eligibility were discussed and resolved. If consensus was not reached, a third reviewer (MFS) was consulted. When data for synthesis or assessment of study quality were insufficient, the authors were contacted by email for clarification, at least twice. If clarification was not obtained, the study was excluded.

The following information was extracted from the included studies: number of subjects, sample characteristics, telerehabilitation characteristics, comparison groups, measured outcomes, duration of follow-ups, and results. The Mendeley reference manager was used to assist with study selection and data extraction.

Two reviewers (FXA and GGS) independently assessed the risk of bias of the included studies using the PEDro scale (Maher et al., 2003; Shiwa et al., 2011). Studies without a clear description of intention-to-treat analysis were considered to not meet this criterion. Lack of description of allocation concealment was inferred from the absence of information on how the allocation list was hidden. Studies without a description of blinding were considered open. Scores below seven were considered to be of low methodological quality (high risk of bias), while scores equal or greater than seven were considered high quality (low risk of bias), consistent with previous studies (Martini et al., 2022; Pinto et al., 2012).

### Certainty of Evidence

Two reviewers (GGS and FXA) independently assessed the certainty of evidence for each outcome using the Grading of Recommendations, Assessment, Development, and Evaluation (GRADE) system (Balshem et al., 2011). GRADE categorizes the certainty of evidence into four levels: (1) high; (2) moderate; (3) low; and (4) very low. The certainty of evidence was downgraded by one level due to (1) limitations in study design (if > 25% of participants were from studies with low methodological quality (PEDro <7)), (2) inconsistency (if the I2 statistic > 50% or when only one study was included in a comparison), and (3) imprecision (if the pooled sample was less than 400 patients in the comparison and/or a single study with less than 400 patients) (Guyatt et al., 2011). Indirect evaluation was not downgraded as patients, interventions, and comparators were similar between comparisons (Pinto et al., 2012). Publication bias was not considered due to the small number of trials in each analysis (<10 studies) (Higgins et al., 2020).

### Data Synthesis and Meta-Analysis

If outcome measures could not be converted to a common numerical scale, a descriptive synthesis was conducted. For quantitative analysis, effect estimates were calculated by comparing the least squares mean percentage change from baseline to the effect at the end of the study for each group (Higgins et al., 2003). For continuous outcomes with consistent units of measurement across studies, results were presented as weighted mean differences with 95% confidence intervals (CIs). Calculations were performed using a random effects method. A p-value ≤ 0.05 was considered statistically significant. The statistical heterogeneity of treatment effects between studies was assessed using Cochran's Q test and the I-square inconsistency test (I2). Values above 25% and 50% were considered indicative of moderate and high heterogeneity, respectively (Higgins et al., 2003). Sensitivity analysis was performed to evaluate the impact of statistical heterogeneity and the review and duration of intervention studies. All analyses were conducted using Review Manager software, version 5.3.

## Results

**Figure 1:**
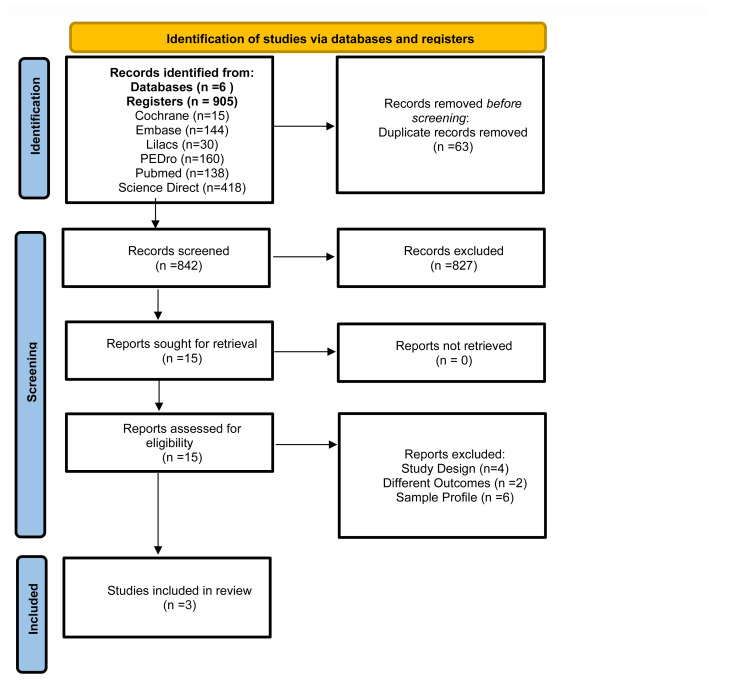
Study Selection and Screening Flowchart

The database search yielded 905 articles, which were screened for eligibility. In the final stage of study screening, 12 studies were excluded: four for being non-randomized clinical trials or other study designs; two due to different outcomes related to physical or functional capacity; and six for including samples with characteristics distinct from traumatic upper limb fractures. After the study selection process, three trials (encompassing a total of 830 patients) were included in this systematic review. This process is illustrated in Figure 1.

All studies included in this systematic review shared certain characteristics: participants with wrist, hand, and finger fractures, no studies involving individuals with proximal upper limb fractures, an adult population, and predominantly female participants in two of the three studies. A summary of these study characteristics is presented in Table 1.

Among the telerehabilitation modalities evaluated, two studies (Blanquero et al., 2020; Suero-Pineda et al., 2023) utilized tablet-based software exercises, while one study (Pech-ArguÓelles et al., 2024) employed app-guided exercise guidance. All telerehabilitation groups participated in a four-week intervention program.

Regarding control groups, two studies (Blanquero et al., 2020; Suero-Pineda et al., 2023) implemented printed exercise booklets, while one study (Pech-ArguÓelles et al., 2024) utilized in-person physical therapy.

To evaluate functional capacity outcomes, the included studies employed similar instruments such as the Disabilities of Arm, Shoulder, and Hand (DASH) questionnaire and the QuickDASH questionnaire. For statistical purposes, DASH scores were converted to QuickDASH scores using the formula: [DASH Score = 1.18 x QuickDASH Score + 3.66] (da Silva et al., 2020). Among the physical capacity outcomes, handgrip strength was the chosen outcome measure by all included studies (Bobos et al., 2020). Another important clinical outcome presented in all studies was pain perception, assessed using the Visual Analogue Scale (VAS). This outcome is directly related to the physical and functional capacity of individuals and was therefore analyzed in the meta-analysis.

No study blinded its participants. However, all studies presented initial comparisons to demonstrate group homogeneity, outcome measures with estimates and variability, and comparisons between groups. The risk of bias score, assessed using the PEDro scale, is presented in Table 2.

### Intervention Effects and Certainty of Evidence

**Figure 2:**
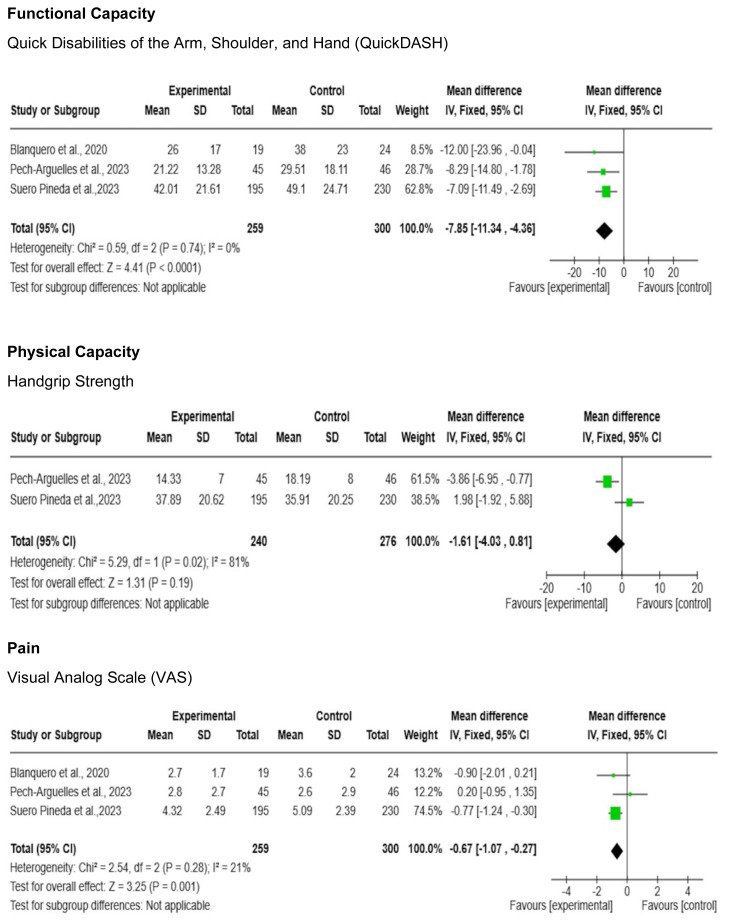
Meta-Analysis of Functional Capacity, Physical Capacity and Pain Outcomes Immediately Post Intervention in a Telerehabilitation vs Control Group

For immediate post-intervention results, there is moderate-quality evidence that telerehabilitation has lower benefits than the control group for physical capacity measured by handgrip strength [MD = 1.75; 95% CI(−0.26, 3.75); I2 = 0%] (Figure 2 and Table 3). However, there is moderate certainty in the evidence that telerehabilitation is favorable to the control group in terms of functional capacity measured by QuickDASH [MD = −7.85; 95% CI(−11.34, −4.36); I2 = 0%] and pain measured by VAS [MD = −0.67; 95% CI(−1.07, −0.27); I2 = 21%] (Figure 2 and Table 3).

**Figure 3:**
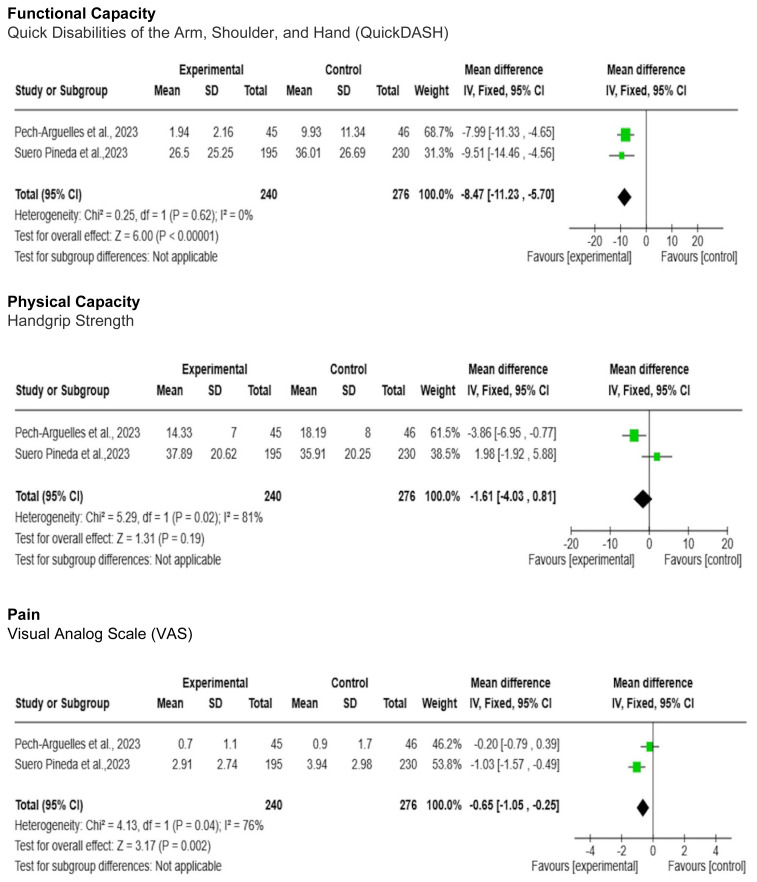
Meta-Analysis of Functional Capacity, Physical Capacity and Pain Outcomes of Medium-Term Follow-Up (2 to 5 months) Post Intervention in a Telerehabilitation vs Control Group

Medium-term follow-up results (2 to 5 months post-intervention) consistently favor telerehabilitation compared to the control group for all analyzed outcomes. It's noteworthy that for handgrip strength [MD = −1.61; 95% CI(−4.03, 0.81); I2 = 81%] and pain [MD = −0.65; 95% CI(−1.05, −0.25); I2 = 76%], there is low certainty of evidence. For QuickDASH [MD = −8.47; 95% CI(−11.23, −5.70); I2 = 0%], there is moderate certainty of evidence (Figure 3 and Table 3).

The outcomes measured immediately after the intervention (Figure 2) and at medium-term follow-up (2 to 5 months) were grouped and analyzed using the meta-analyses presented below:The outcomes measured immediately after the intervention (Figure 2) and medium-term follow-up (2 to 5 months) after the intervention (Figure 3), which could be grouped, were analyzed using meta-analyses presented below:

It should be noted that for statistical purposes, baseline values were considered prior to the initiation of telerehabilitation treatment. Final values refer to the immediate post-intervention period, following the conclusion of the last treatment session, respecting the duration of each protocol. Due to varying follow-up periods among the studies, a timeframe of 2 to 5 months after the end of the intervention was considered. For a more comprehensive and coherent interpretation of these results, an analysis associated with the assessment of evidence certainty is necessary. To this end, the same outcomes from the meta-analyses were evaluated using the GRADE system. The results are presented in Table 3.

Given the presented meta-analysis, results can be interpreted for two distinct periods: immediately after the intervention and during medium-term follow-up (2 to 5 months post-intervention). In the initial phase, telerehabilitation demonstrated favorable outcomes for functional capacity (QuickDASH) and pain perception (VAS), but was less effective than the control group in terms of physical capacity (handgrip strength). In the medium-term follow-up, telerehabilitation consistently yielded favorable results for all evaluated outcomes.

## Discussion

This study aimed to systematically review the scientific evidence to identify the effects of telerehabilitation on the physical and functional capacity of individuals with traumatic upper limb fractures. Based on the three included randomized clinical trials, it's clear that for an adult population, predominantly female (in two of the three studies) and with wrist, hand, and finger fractures, there is moderate certainty of evidence that telerehabilitation had favorable effects on functional capacity (QuickDASH) and pain (VAS) immediately after the intervention. However, it exhibited worse effects than the control group in terms of physical capacity (measured by handgrip strength).

During medium-term follow-up (2 to 5 months post-intervention), telerehabilitation demonstrated favorable effects on all evaluated outcomes, with low certainty of evidence for physical capacity and pain, and moderate certainty for functional capacity.

To our knowledge, this is the first systematic review investigating the effects of telerehabilitation on the physical and functional capacity of patients with traumatic upper limb fractures. We prospectively registered the review protocol, conducted a comprehensive search of electronic databases, and provided justifications for excluding individual studies. This review adhered to PRISMA recommendations, determined evidence certainty using the GRADE framework, and fulfilled all critical items proposed by the AMSTAR 2 checklist (Shea et al., 2017). The low heterogeneity of the included studies and high methodological quality (low risk of bias) of two of the three studies are strengths of this research.

Some limitations of this study include: the small number of studies and participants; per PEDro evaluation, none of the group assignments for studies were blinded to the investigators; some of the confidence intervals for studies included zero, which should be regarded as a limitation. However, our comprehensive search strategy makes it less likely that any trials were missed, especially considering that two of the three studies were very recent.

In 2017, there were approximately 18 million hand and wrist fractures worldwide. Although the rate of these injuries is decreasing in countries with higher socioeconomic development, regions with lower and middle socioeconomic indices have experienced an increasing rate of hand injuries over the past 27 years (Crowe et al., 2020). A meta-analysis showed that during the COVID-19 pandemic, the number of fractures has decreased, but there was a higher mortality rate associated with fractures (Lim, Ridia & Pranata, 2021).

While this review focused on upper limb fractures, all included studies involved patients with wrist, hand, and finger fractures, adults, and predominantly females. In two (Suero-Pineda et al., 2023; Pech-Arguelles et al.,2023) of the three studies included in the review, there was a higher prevalence of women in both the intervention group and the control group. A Swedish study with 23,917 individuals sustained 27,169 fractures 64.5% of the fractures occurred in women and the five most common fractures accounted for more than 50% of all fractures: distal radius, proximal femur, ankle, proximal humerus, and metacarpal fractures (Bergh et al.,2020). Over the age of 60 years, females were 2.3 times more likely to sustain a fracture than males (Singer et al.,1998). Unlike the present study, males are the majority of those who suffer hand and wrist fractures (incidence ratio of 1.8:1 between men and women) (Crowe et al., 2020). In a Chinese study to identify the epidemiological characteristics of traumatic fractures during the COVID-19 pandemic, 2489 patients and a total of 2590 fractures were included, finding a higher prevalence of men compared to women (Lv et al.,2020). For an American epidemiological study, the prevalence of wrist fractures was higher in men than in women aged between 50 and 60 years old, but higher in women than in men aged with 60 or more (Yie, Li & Nie, 2022).

Telerehabilitation emerges as an innovative and technological alternative that provides access to rehabilitation programs and protocols, fostering greater autonomy and encouraging self-care. Various musculoskeletal conditions have benefited from different telerehabilitation modalities, whether synchronous, asynchronous, through applications, or software. Some modalities can be as effective as conventional treatment, offering advantages such as reducing the number of in-person consultations and promoting safety and motivation in exercise prescription and performance (Phang et al., 2023). Several systematic reviews have demonstrated that telerehabilitation can be beneficial for the physical and functional capacity of individuals with knee (Piqueras et al., 2013) and hip (Magaziner et al., 2000) arthroplasty.

The evidence presented provides a foundation and assurance for the telerehabilitation modalities used in the included studies: rehabilitation programs delivered through mobile applications and software. The intervention time of the included studies was consistent at four weeks, and the maximum follow-up time was five months. This uniformity may have contributed to similar results for functional capacity and pain, both immediately after the intervention and during medium-term follow-up (2 to 5 months). Physical capacity exhibited contrasting results during these two analysis periods: the control group demonstrated immediate benefits, while the telerehabilitation group showed superiority in the medium term. Evidence suggests that the type of intervention and the manner in which telerehabilitation is implemented do not significantly influence the outcomes, and modality choice should align with user preferences and satisfaction (Baroni et al., 2023).

The potential impact of the control group's interventions on immediate post-intervention handgrip strength gains cannot be overlooked. One study employed in-person care for two consecutive weeks, while others prescribed exercises via printed booklets. Both modalities, while potentially facilitating rapid adaptation and early results, present limitations. The group receiving in-person care, upon cessation of treatment, may have experienced a decline in strength and functional gains during follow-up assessments (2 to 5 months post-intervention). Similarly, while printed booklets offer accessibility, their long-term adherence may be compromised, potentially contributing to lower handgrip strength outcomes at follow-up.

The reviewed studies utilized a 4-week telerehabilitation intervention, necessitating consideration of technology adaptation and treatment adherence timelines. While a 30-day telerehabilitation program proved effective in improving self-efficacy, mobility, quality of life, and patient satisfaction in elderly hip fracture patients (Bedra & Filkenstein, 2015), an 8-week program was required to significantly enhance pain, function, quality of life, kinesiophobia, satisfaction, and motivation in chronic low back pain patients (Özden et al., 2022).

The meta-analysis findings on handgrip strength may reflect a potential delay in telerehabilitation group adaptation to technology, resulting in more pronounced effects during the medium-term follow-up (2 to 5 months).

Moreover, the assessment instruments utilized in the included studies provide a degree of confidence regarding the research outcomes, as they likely reflect the reality of the clinical conditions of the study samples. For example, a previous systematic review analyzing 898 studies on virtual physical therapy assessments for musculoskeletal disorders found good validity and excellent reliability for pain, muscle strength, and functional capacity (Mani et al., 2017).

Regarding functional capacity outcomes, both the control and telerehabilitation groups experienced improvements in QuickDASH scores, with greater benefits observed in the intervention group as confirmed by the meta-analysis. QuickDASH is a validated assessment instrument widely used in scientific literature to measure shoulder, arm, and hand disabilities (Bobos et al., 2020).

For physical capacity, both the control and telerehabilitation groups demonstrated improvements in handgrip strength indices, with greater improvements observed in the intervention group. However, this statistical difference was confirmed by the meta-analysis only for the medium term (2 to 5 months post-intervention). The handgrip strength assessment instrument used in this systematic review is widely recognized in the scientific literature as a reliable and valid procedure among healthy participants and in various clinical populations (Shea et al., 2017).

## Conclusion

The results of this systematic review offer optimism for physiotherapists considering telerehabilitation as a treatment option for patients with traumatic upper limb fractures. Although there has been an increase in clinical trials, additional studies with high methodological quality and strong evidence are needed to solidify confidence in the use of this modality for improving physical condition and functional capacity in this patient population.

This systematic review demonstrated that for adults with traumatic wrist, hand, and finger fractures, there is some evidence that telerehabilitation has superior outcomes to the control group in terms of functional capacity (measured by DASH) and pain perception (measured by VAS) immediately after the intervention. Conversely, during the same period, physical capacity (measured by handgrip strength) exhibited better results for the control group with moderate certainty of evidence. Medium-term results (2 to 5 months post-intervention) indicate favorable effects for the telerehabilitation group in all measured outcomes, including physical capacity and pain (low certainty of evidence) and functional capacity (moderate certainty of evidence)

## Figures and Tables

**Table 1:** Data Extracted from the Three Articles Included in the Systematic Review

Study	Sample Characteristics	Intervention Group (Telerehabilitation)	Comparison Group (Control)	Measured Outcomes	Results (IG x CG)
Blanquero et al., 2020 Origin: Spain Language: English	18 + Local: -Wrist -Hand -Fingers Fractures + Soft Tissue Injuries	*n=40* Women: 32% ***Intervention*** Duração: 4 w. Modality: - Software (Tablet)	*n=34* Women: 44% ***Intervention*** Duração: 4 w. Modality: - Printed booklets	**Final outcome measure:** 4 w ***Functional Capacity:*** - QuickDASH; ***Physical Capacity*:** - Pinch Force - Grip Strength Pain	**QuickDASH** IG > CG a **Pinch Force** IG > CG a **Handgrip Strength** IG > CG a **Pain** IG > CG a
Suero-Pineda et al., 2023 Origin: Spain Language: English	18 + Local: -Wrist -Hand -Fingers Fractures + Soft Tissue Injuries	*n=270* Women: 68% ***Intervention*** Duração: 4 w. Modality: - Software (Tablet)	*n=393* Women: 66% ***Intervention*** Duração: 4 w. Modality: - Printed booklets	**Final outcome measure::** 4 w. **Follow-up:** 2 m Post Intervention. ***Functional Capacity:*** - QuickDASH; - PRWE ***Physical Capacity*:** - Handgrip Strenght **Pain**	**QuickDASH** IG > CG b **PRWE** IG > CG b **Handgrip Strenght** IG > CG b **Pain** IG > CG b
Pech-Arguelles et *al.*, 2023 Origin: México Language: Spanish	18 + Local: - Wrist Fractures	*n=45* Women: 55,6% ***Intervention*** Duration: 4 w Modality: -Application	*n=48* Women: 58,7% ***Intervention*** Duration: 2 weeks Modality: -In-person service	**Final outcome measure:** 4 w. **Follow-up:** 5 m Post Intervention. ***Functional Capacity:*** - DASH ***Physical Capacity*:** - ROM; - Handgrip Strenght ***Pain:*** **Quality of Life** *- SF-36*	**DASH** *- Intragroups:* IG > CG b - *Intergroups:* IG = CG **ROM** *- Intragroups***:** IG > CG^b^ - *Intergroups:* IG = CG **Handgrip Strenth** **-** *Intragroups:* IG > CG^b^ - *Intergroups:* IG = CG **Pain** **-** *Intragroups:* IG > CG b - *Intergroups:* IG = CG **Quality of Life** *- Intragroups:* IG > CG b - *Intergroups:* IG = CG

*Note.* IG: Intervention Group: Telerehabilitation Group; CG: Control Group;

a = Inaccurate results due to wide confidence interval.

PRWE: Patient Rated Wrist Evaluation;

b statistically significant difference with analysis of variance, considering a statistical significance level of 95% (p≤0.05);

DASH = Disabilities of Arm, Shoulder and Hand Questionnaire; ROM: Range of Motion; m: months; w: weeks; SF-36: Short Form 36 items – Quality of Life Questionnaire. VAS: Visual Analogue Pain Scale

**Table 2:** Risk of Bias Score (PEDro Scale)

Study	1*	2	3	4	5	6	7	8	9	10	11	Score
Blanquero et al., 2022	Y	Y	Y	Y	N	Y	Y	Y	Y	Y	Y	9/10
Suero-Pineda et al., 2023	Y	N	N	Y	N	N	Y	Y	N	Y	Y	5/10
Pech-Arguelles et al., 2023	Y	Y	Y	Y	N	N	Y	Y	Y	Y	Y	8/10

*Note*. Y= Yes; N = No; 1*: eligibility criteria and source of participants, does not contributing to the total score; 2: random allocation;3: concealed allocation; 4: baseline comparability; 5: blinded participants; 6: blinded therapists; 7: blinded assessors; 8: adequate follow up; 9: intention-to-treat analysis; 10: between group comparison; 11: point estimates and variability.

**Table 3:** Summary of Findings and Certainty of Evidence (GRADE)

N (Study Design)	Risk of Bias	Inconsistency	Indirectness	Imprecision	Publication Bias	Outcome	Total of Participants	MD (CI 95%); I^2^	Certainty of Evidence
**Functional Capacity (Telerehabilitation x Control)**									
3 (RCT)	Serious^a^	Not Serious	Not Rated	Not Serious	NA	HS	559	1.75 (−0.26, 3.75); 0%	Moderated
*Follow Up – Medium Term (2 to 5 months)*									
2 (RCT)	Serious^a^	Serious^b^	Not Rated	Not Serious	NA	HS	516	−1.61 (−4.03, 0.81); 81%	Low
**Physical Capacity (Telerehabilitation x Control)**									
3 (RCT)	Serious^a^	Not Serious	Not Rated	Not Serious	NA	QuickDASH	559	−7.85 (−11.34, −4.36); 0%	Moderated
*Follow Up – Medium Term (2 to 5 months)*									
2 (RCT)	Serious^a^	Not Serious	Not Rated	Not Serious	NA	QuickDASH	516	−8.47 (−11.23, −5.70); 0%	Moderated
**Pain (Telerehabilitation x Control)**									
3 (RCT)	Serious^a^	Not Serious	Not Rated	Not Serious	NA	VAS	559	−0.67 (−1.07, −0.27); 21%	Moderated
*Follow Up – Medium Term (2 to 5 months)*									
2 (RCT)	Serious^a^	Serious^b^	Not Rated	Not Serious	NA	VAS	516	−0.65(−1.05, −0.25); 76%	Low

*Note*: CI 95%: Confidence Interval 95%: RCT: Randomised Clinical Trial; NA: Not applicable; HS: Handgrip Strength; VAS: Visual Analogic Scale

Reason for Reducing 1 Level of Certainty of Evidence:

a: More than 25% of the participants came from studies with low methodological quality (PEDRO Score < 7)

Certainty of Evidence: Low (2/4) - Our confidence in the effect estimate is limited: The true effect may be substantially different from the estimate of the effect; Moderated (3/4) - We are moderately confident in the effect estimate: The true effect is likely to be close to the estimate of the effect, but there is a possibility that it is substantially different.

## References

[b1-ijt-17-1-6667] Balshem H Helfand M Schünemann HJ Oxman AD Kunz R Brozek J Vist GE Falck-Ytter Y Meerpohl J Norris S 2011 GRADE guidelines: 3. Rating the quality of evidence Journal of Clinical Epidemiology 64 4 401 406 10.1016/j.jclinepi.2010.07.015 21208779

[b2-ijt-17-1-6667] Baroni MP Jacob MFA Rios WR Fandim JV Fernandes LG Chaves PI Fioratti I Saragiotto BT 2023 The state of the art in telerehabilitation for musculoskeletal conditions Archives of Physiotherapy 13 1 10.1186/s40945-022-00155-0 PMC9810517 36597130

[b3-ijt-17-1-6667] Bedra M Finkelstein J 2015 Feasibility of post-acute hip fracture telerehabilitation in older adults Studies in Health Technology and Informatics 210 469 473 25991191

[b4-ijt-17-1-6667] Bergh C Wennergren D Möller M Brisby H 2020 Fracture incidence in adults in relation to age and gender: A study of 27,169 fractures in the Swedish Fracture Register in a well-defined catchment area PLOS ONE 15 12 e0244291 10.1371/journal.pone.0244291 33347485 PMC7751975

[b5-ijt-17-1-6667] Blanquero J Cortés-Vega MD Rodríguez-Sánchez-Laulhé P Corrales-Serra BP Gómez-Patricio E Díaz-Matas N Suero-Pineda A 2020 Feedback-guided exercises performed on a tablet touchscreen improve return to work, function, strength and healthcare usage more than an exercise program prescribed on paper for people with wrist, hand or finger injuries: A randomised trial Journal of Physiotherapy 66 4 236 242 10.1016/j.jphys.2020.09.012 33069608

[b6-ijt-17-1-6667] Bobos P Nazari G Lu Z MacDermid JC 2020 Measurement properties of the hand grip strength assessment: A systematic review with meta-analysis Archives of Physical Medicine and Rehabilitation 101 3 553 565 10.1016/j.apmr.2019.10.183 31730754

[b7-ijt-17-1-6667] Bucki FM Clay MB Tobiczyk H Green BN 2021 Scoping review of telehealth for musculoskeletal disorders: Applications for the COVID-19 pandemic Journal of Manipulative and Physiological Therapeutics 44 7 558 565 10.1016/j.jmpt.2021.12.003 35249750 PMC8892222

[b8-ijt-17-1-6667] Carey JR Durfee WK Bhatt E Nagpal A Weinstein SA Anderson KM Lewis SM 2007 Comparison of finger tracking versus simple movement training via telerehabilitation to alter hand function and cortical reorganization after stroke Neurorehabilitation and Neural Repair 21 3 216 232 10.1177/1545968306292381 17351083

[b9-ijt-17-1-6667] Cottrell MA Galea OA O’Leary SP Hill AJ Russell TG 2017 Real-time telerehabilitation for the treatment of musculoskeletal conditions is effective and comparable to standard practice: A systematic review and meta-analysis Clinical Rehabilitation 31 5 625 638 10.1177/0269215516645148 27141087

[b10-ijt-17-1-6667] Cottrell MA Russell TG 2020 Telehealth for musculoskeletal physiotherapy Musculoskeletal Science and Practice 48 102193 10.1016/j.msksp.2020.102193 32560876 PMC7261082

[b11-ijt-17-1-6667] Crowe CS Massenburg BB Morrison SD Chang J Friedrich JB Abady GG Alahdab F Alipour V Arabloo J Asaad M Banach M Bijani A Borzì AM Briko NI Castle CD Cho DY Chung MT Daryani A Demoz GT James SL 2020 Global trends of hand and wrist trauma: a systematic analysis of fracture and digit amputation using the Global Burden of Disease 2017 Study Injury Prevention 26 Suppl 2 i115 i124 10.1136/injuryprev-2019-043495 32169973 PMC7571361

[b12-ijt-17-1-6667] da Silva NC Chaves TC dos Santos JB Sugano RMM Barbosa RI Marcolino AM Mazzer N Fonseca MCR 2020 Reliability, validity and responsiveness of Brazilian version of QuickDASH Musculoskeletal Science and Practice 48 102163 10.1016/j.msksp.2020.102163 32560867

[b13-ijt-17-1-6667] Guyatt GH Oxman AD Kunz R Brozek J Alonso-Coello P Rind D Devereaux PJ Montori VM Freyschuss B Vist G Jaeschke R Williams JW Murad MH Sinclair D Falck-Ytter Y Meerpohl J Whittington C Thorlund K Andrews J Schünemann HJ 2011 GRADE guidelines 6. Rating the quality of evidence - Imprecision Journal of Clinical Epidemiology 64 12 1283 1293 10.1016/j.jclinepi.2011.01.012 21839614

[b14-ijt-17-1-6667] Handoll HHG Elliott J Thillemann TM Aluko P Brorson S 2022 Interventions for treating proximal humeral fractures in adults Cochrane Database of Systematic Reviews 2022 6 10.1002/14651858.CD000434.pub5 PMC9211385 35727196

[b15-ijt-17-1-6667] Higgins JP Thompson SG Deeks JJ Altman DG 2003 Measuring inconsistency in meta-analyses BMJ (Clinical research edition) 327 7414 557 560 10.1136/bmj.327.7414.557 PMC192859 12958120

[b16-ijt-17-1-6667] Higgins JPT Green S Ben Van Den A 2020 Cochrane Handbook for Systematic Reviews of Interventions 15 International Coaching Psychology Review

[b17-ijt-17-1-6667] Liberati A Altman DG Tetzlaff J Mulrow C Gøtzsche PC Ioannidis JPA Clarke M Devereaux PJ Kleijnen J Moher D 2009 The PRISMA statement for reporting systematic reviews and meta-analyses of studies that evaluate health care interventions: *Explanation and Elaboration* Journal of Clinical Epidemiology 62 10 e1 e34 10.1016/j.jclinepi.2009.06.006 19631507

[b18-ijt-17-1-6667] Lim MA Ridia KGM Pranata R Epidemiological pattern of orthopaedic fracture during the COVID-19 pandemic: A systematic review and meta-analysis 2021 Journal of Clinical Orthopaedics & Trauma 16 16 23 10.1016/j.jcot.2020.12.028 33398227 PMC7773000

[b19-ijt-17-1-6667] Lv H Zhan Q Yin Y Zhu Y Wang J Hou Z Zhang Y Chen W 2020 Epidemiologic characteristics of traumatic fractures during the outbreak of coronavirus disease 2019 (COVID-19) in China: A retrospective & comparative multi-center study Injury 51 8 1698 1704 10.1016/j.injury.2020.06.022 32563519 PMC7295526

[b20-ijt-17-1-6667] Magaziner J Hawkes W Hebel JR Zimmerman SI Fox KM Dolan M Felsenthal G Kenzora J 2000 Recovery from hip fracture in eight areas of function Journal of Gerontology 55 9 M498 M507 https://academic.oup.com/biomedgerontology/article/55/9/M498/2948037 10.1093/gerona/55.9.m498 10995047

[b21-ijt-17-1-6667] Maher CG Sherrington C Herbert RD Moseley AM Elkins M 2003 Reliability of the PEDro scale for rating quality of randomized controlled trials Physical Therapy 83 8 713 721 http://www.ncbi.nlm.nih.gov/pubmed/12882612 12882612

[b22-ijt-17-1-6667] Mani S Sharma S Omar B Paungmali A Joseph L 2017 Validity and reliability of Internet-based physiotherapy assessment for musculoskeletal disorders: A systematic review Journal of Telemedicine and Telecare 23 3 379 391 10.1177/1357633X16642369 27036879

[b23-ijt-17-1-6667] Martini JD Ferreira GE Xavier de Araujo F 2022 Pilates for neck pain: A systematic review and meta-analysis of randomised controlled trials Journal of Bodywork and Movement Therapies 31 37 44 10.1016/j.jbmt.2022.03.011 35710219

[b24-ijt-17-1-6667] Özden F Sarı Z Karaman ÖN Aydoğmuş H 2022 The effect of video exercise-based telerehabilitation on clinical outcomes, expectation, satisfaction, and motivation in patients with chronic low back pain Irish Journal of Medical Science 191 3 1229 1239 10.1007/s11845-021-02727-8 34357527

[b25-ijt-17-1-6667] Pech-Arguelles RC Miranda-Ortiz YJ Velázquez-Hernández HE Domínguez-Cordero R Ruiz-Pacheco C Figueroa-García J Rojano-Mejía D 2024 Tele-rehabilitation program in patients with distal radius fracture: a controlled clinical trial Cirugía y Cirujanos (English Edition) 92 1 10.24875/cirue.m22000599 37156230

[b26-ijt-17-1-6667] Peretti A Amenta F Tayebati SK Nittari G Mahdi SS 2017 Telerehabilitation: Review of the state-of-the-art and areas of application JMIR Rehabilitation and Assistive Technologies 4 2 e7 10.2196/rehab.7511 28733271 PMC5544892

[b27-ijt-17-1-6667] Phang JK Lim ZY Yee WQ Tan CYF Kwan YH Low LL 2023 Post-surgery interventions for hip fracture: A systematic review of randomized controlled trials BMC Musculoskeletal Disorders 24 1 10.1186/s12891-023-06512-9 PMC10210378 37231406

[b28-ijt-17-1-6667] Pinto RZ Maher CG Ferreira ML Hancock M Oliveira VC McLachlan AJ Koes B Ferreira PH 2012 Epidural corticosteroid injections in the management of sciatica Annals of Internal Medicine 157 12 865 10.7326/0003-4819-157-12-201212180-00564 23362516

[b29-ijt-17-1-6667] Piqueras M Marco E Coll M Escalada F Ballester A Cinca C Belmonte R Muniesa JM 2013 Effectiveness of an interactive virtual telerehabilitation system in patients after total knee arthroplasty: A randomized controlled trial Journal of Rehabilitation Medicine 45 4 392 396 10.2340/16501977-1119 23474735

[b30-ijt-17-1-6667] Robinson LS Sarkies M Brown T O’Brien L 2016 Direct, indirect and intangible costs of acute hand and wrist injuries: A systematic review Injury 47 12 2614 2626 10.1016/j.injury.2016.09.041 27751502

[b31-ijt-17-1-6667] Shea BJ Reeves BC Wells G Thuku M Hamel C Moran J Moher D Tugwell P Welch V Kristjansson E Henry DA 2017 AMSTAR 2: A critical appraisal tool for systematic reviews that include randomised or non-randomised studies of healthcare interventions, or both BMJ (Online) 358 10.1136/bmj.j4008 PMC5833365 28935701

[b32-ijt-17-1-6667] Shiwa SR Costa LOP da Costa LCM Moseley A Hespanhol LC Junior Venâncio R Ruggero C de Sato TO Lopes AD 2011 Reproducibility of the Portuguese version of the PEDro Scale Cadernos de Saúde Pública 27 10 2063 2068 10.1590/S0102-311X2011001000019 22031210

[b33-ijt-17-1-6667] Singer BR McLauchlan GJ Robinson CM Christie J 1998 Epidemiology of fractures in 15, 000 adults Journal of Bone & Joint Surgery British Volume 80-B 2 243 248 10.1302/0301-620X.80B2.0800243 9546453

[b34-ijt-17-1-6667] Suero-Pineda A Oliva-Pascual-Vaca Á Durán MRP Sánchez-Laulhé PR García-Frasquet MÁ Blanquero J 2023 Effectiveness of a telerehabilitation evidence-based tablet app for rehabilitation in traumatic bone and soft tissue injuries of the hand, wrist, and fingers Archives of Physical Medicine and Rehabilitation 104 6 932 941 10.1016/j.apmr.2023.01.016 36758713

[b35-ijt-17-1-6667] Wang Q Hunter S Lee RL-T Chan SW-C 2023 The effectiveness of a mobile application-based programme for rehabilitation after total hip or knee arthroplasty: A randomised controlled trial International Journal of Nursing Studies 140 104455 10.1016/j.ijnurstu.2023.104455 36821950

[b36-ijt-17-1-6667] Ye J Li Q Nie J (2022) Prevalence, characteristics, and associated risk factors of wrist fractures in Americans above 50: The cross-sectional NHANES study Frontiers in Endocrinology 13 10.3389/fendo.2022.800129 PMC9082306 35547001

